# Negotiating an academic start-up package and job offer as an incoming tenure-track professor in the life sciences

**DOI:** 10.1186/s12919-025-00327-3

**Published:** 2025-05-16

**Authors:** Leslie A. Caromile, Verónica A. Segarra

**Affiliations:** 1 Center for Vascular Biology, UConn Health, Farmington, CT 06030 USA; 2 American Society for Cell Biology, Rockville, MD 20852 USA; 3 Department of Biological Sciences, Goucher College, Baltimore, MD 21204 USA; 4 Department of Chemistry, Goucher College, Baltimore, MD 21204 USA

**Keywords:** Start-up package, Academic start-up package, Faculty job offer, Tenure-track job offer, Faculty job offer negotiation, Academic job offer negotiation, Negotiation

## Abstract

Start-up packages were once predominantly associated with research-intensive institutions. However, they are now increasingly important for teaching-focused and primarily undergraduate institutions due to rising expectations for research involvement. Effective negotiation for tenure-track start-up packages is essential for aligning the candidates' and institutions' interests and goals. Candidates must understand the various components of an offer, such as start-up funds, teaching loads, research expectations, and tenure requirements, to negotiate terms that align with their needs and goals. However, navigating these negotiations can be daunting, particularly for individuals from historically underserved and excluded groups (HUE) in STEM fields who may lack access to supportive networks. In this article, we aim to provide a detailed guide on how to negotiate an effective tenure-track start-up package. We emphasize that this process can be beneficial for both the candidate and the institution, as it helps ensure that their values and goals are aligned, ultimately increasing the chances of success for both parties. This article builds upon our earlier publications and provides comprehensive guidance for negotiating strategies.

## Background

It is commonly recommended that candidates seeking faculty positions should have a precise understanding of the terms of the offer from an institution prior to accepting the appointment. However, this objective can be challenging to achieve as candidates may not possess the requisite knowledge to negotiate a job offer's various elements and parameters [1, 2]. It is imperative for candidates to be aware of the different components of an offer, including start-up funds, teaching loads, research expectations, and tenure requirements. Acquiring this knowledge will empower the candidate to negotiate effectively and obtain the start-up package that affords them the resources, support, and autonomy necessary to thrive in their new role and be well on their way to securing tenure.

One key element of a faculty job offer is the start-up package. The startup package is a collection of resources an institution provides an incoming faculty member to start their research program. Start-up packages have traditionally been considered essential by research-intensive institutions, given the focus on research among faculty. However, these resources are also becoming increasingly important for teaching-focused institutions and primarily undergraduate institutions (PUIs). In today's academic environment, faculty members at PUIs are increasingly expected to engage in research activities that involve undergraduate students.

Institutions are best served by prioritizing the success of their incoming faculty members. Therefore, they should make every effort to provide clear and concise information about the terms of an offer to ensure that candidates are positioned to succeed in their new academic community. Unfortunately, candidates are often left to navigate the terms and conditions of an offer independently, which can be challenging and overwhelming.

Candidates with a supportive mentoring network are more likely to receive the guidance they need to navigate and negotiate a competitive startup package successfully. Research suggests that people who belong to groups that have been historically underserved and excluded (HUE) in STEM fields have less access to such supportive networks compared to their well-represented peers [1, 2]. This mentoring gap disproportionately affects the ability of historically minoritized individuals in STEM to negotiate a robust start-up package that will allow them a strong start in academia, potentially further contributing to disparities in tenure success. In addition to mentoring gaps, there is implicit bias that candidates face in academia and during the hiring process, especially HUEs [3–5]. Content that openly discusses the unwritten rules of the academy and the degree to which an institution's investment (i.e., details of start-up package) impacts a candidate’s chances for success can help close these mentoring gaps.

Here we aim to build upon our previous publications, which briefly mentioned job negotiation and start-up packages as components of securing a faculty position [6–8] titled "Accomplishing Career Transitions (ACT) 2019: Professional Development for Postdocs and Tenure-track Junior Faculty in the Biomedical Sciences", which was published by the American Society for Cell Biology (ASCB) in 2021 [8]. Although the NIH/NIGMS Innovative Programs to Enhance Research Training funded ACT program, a professional development program established and managed by the ASCB, aims to provide support to HUE postdocs and tenure-track junior faculty in STEM fields, the principles discussed in this article are broadly applicable to all and are geared towards individuals who have landed their first faculty position and those who have decided to move institutions into new faculty appointments. We aim to promote open discussions about effective negotiation strategies for tenure-track start-up packages. By doing so, we hope to emphasize this negotiation as an opportunity for both the faculty candidate and the institution to ensure that their values and goals align with one another.

### Overview of how to negotiate a job offer: the offer negotiation process

While the exact negotiation process often varies depending on the institution, it will likely involve a combination of verbal (in-person, phone, or videoconference) and written communications, both informal (e-mail) and formal (revisions to the offer letter).

Generally, the offer letter will not arrive unannounced but rather follow official communications clearly indicating that the institution wishes to make an offer. These communications between the candidate and the institution representative who has been charged with negotiating on the institution’s behalf in an official capacity. The institution representative can be a department chair, dean, or provost, depending on the size of the academic institution. It is important to note that informal expressions of optimism by a department member or the search committee chair regarding an individual's fit for the department and institution do not guarantee a job offer.

During the communications that precede the arrival of the offer letter, the institution’s representative will want to ascertain the elements needed to make the candidate a competitive offer in writing. During these conversations, it is important that the candidate share what are the parameters they would ideally want included in the offer letter. It is unlikely that the institution will be able to include all items in the candidate’s wish list. The candidate should therefore consider selecting one to three top-priority items to negotiate and expect that some, but not all, of the requests will be granted. This is where negotiation ensues. For this reason, it is normal and acceptable to request revisions to an offer letter before signing–it is not unusual for an offer to go through two or three drafts before it is accepted. This will ensure that the offer letter reflects the needs of the candidate and what they need to be set up for success in their faculty position. Candidates should be professional and always negotiate respectfully, truthfully, and in good faith. Candidates should be prepared to provide clear, solid scientific or professional justification for what they request, including the reasoning behind counteroffers. Candidates should also prepare to ask insightful questions that can help inform their decision-making. This is where preparing for negotiating becomes most important.

If the candidate has competing offers at hand, they can request their interlocutor (chair, dean, or provost–authorized institutional representative) to match the offers if they are better in terms of salary, start-up money, or other benefits of interest. In these cases, the candidate should be prepared to provide copies of competing offers as requested.

Throughout the process, a number of resources exist for getting advice on job negotiations, such as Future/New PI Slack channels (https://futurepislack.wordpress.com/; https://newpislack.wordpress.com/), university career services (some of which have open access content online), colleagues in the candidate's same fellowship or professional development program, and scientific societies. Publications such as books [12] and articles containing case studies or advice (https://medium.com/@jasoncorso/how-to-negotiate-your-first-faculty-job-offer-852beed1dd73; https://bmatb.medium.com/negotiating-faculty-jobs-7b3b0d88aec3; https://www.niehlab.com/getting-a-faculty-job; https://hbr.org/2014/04/15-rules-for-negotiating-a-job-offer) will likely be useful as well.

### Prepare for the process: doing the homework

It is helpful for the candidate to have an idea of whether the items on their wish list are within reach at the institution of interest. For example, if the amount of start-up funds they would like is 4 or 5 times what is usually given to incoming faculty, it would be unrealistic to expect the institution to automatically grant the requested funds without trying to negotiate a lower amount. In this way, it will be important for the candidate to do their homework or research the expected or reasonable values for the faculty position and institution being considered.

There are additional benefits to the candidate preparing the needed inventory of laboratory equipment well before the interview. For example, having this list would enable them to appraise the department's resources—for instance, during a tour—and preliminarily assess what needs may need to be addressed during the negotiation.

While some institutions include the salary range in the faculty job announcement, this is not common practice. There are several resources available online that you can use to estimate these values. For example, data reports from the Faculty Compensation Survey administered by the American Association of University Professors (AAUP) are available on the AAUP website (https://www.aaup.org/our-work/research/FCS). Moreover, if you are interested in faculty salary information from public institutions, those are readily available open access on many sites online. Faculty candidates are rarely asked to name a desired salary as part of their wish list, so preparing to name a number is likely not necessary. As part of the negotiations, candidates can request an increase in salary from the initial draft offer if they have clear professional and/or personal justification. At the same time, institutions may have constraints that prevent them from offering higher salaries (e.g., internal pay equity rules; salary bands created by statute, in the case of public schools). Sometimes, candidates can negotiate a one-time signing bonus (4–5 figures) in lieu of a higher salary. A higher base salary is more desirable than a one-time bonus, but institutions may be more able to provide the latter than the former.

Another consideration is that compensation and benefits can come in many forms. These, too, can be negotiated. For example, other forms of compensation include tuition reimbursement, daycare, and professional development funds. Be flexible when considering these elements as part of what you are negotiating. Part of doing your homework is to think about to what extent you are willing to include this as part of the negotiation process.

Depending on the institution, especially those focusing on teaching, overload pay for accepting additional teaching responsibilities during a term might be available. Candidates should consider this type of opportunity carefully, as taking on too many responsibilities can be overwhelming, especially for a brand-new faculty member. It is essential to ensure that the quality of instruction and the commitment to primary employment obligations are not compromised when accepting additional responsibilities.

Finally, it is important for the candidate to carefully read the faculty handbook and the appropriate guidelines (departmental, school, or disciplinary) related to tenure and promotion. If you have a joint appointment, you must understand how you will be evaluated and if the same guidelines apply to each department. A clear understanding of these guidelines will help you ask for everything you need to succeed.

### There is no one-size-fits-all start-up package

While every start-up package aims to provide a tenure-track faculty member with the necessary resources to succeed in their research program, there is no one-size-fits-all approach to creating such a package. The resources offered must align with the individual's vision for their research program. These resources should not only help them publish their first few scientific papers but also generate data that fosters new ideas and serves as preliminary data to obtain external funds to sustain research beyond the end of the start-up resources. This highlights the importance of a candidate's well-defined vision and goals for their future research program. The better defined their vision and goals, the better position they will be in to advocate for the resources they need to meet these goals. Therefore, it is important for candidates to start creating their vision as early as possible.

### Start preparing early: inventorying and listing the resources needed

Concurrent with creating a vision for your research program, you should be building a detailed list of what you will need to achieve it–from instruments and consumables to space and collaborators. It is recommended to start building this list early. This list should be specific and include suppliers and catalog numbers if there are strong preferences, especially when related to major or specialized equipment (e.g., microscopes, mass specs), allowing for time savings in the future and facilitating access to all that is needed. If your list also includes estimated prices, the total of these needs can be added to generate an estimated dollar amount to get your lab up and running. Please keep in mind that once an offer is made, the institution will likely ask for a list of instrumentation and materials that are needed. Table 1 lists items and areas that can be negotiated as part of a start-up package for the reader to consider. Additional considerations can be found in relevant articles published in the previous BMC ASCB ACT Proceedings issue [6, 7, 9, 10].

**Table 1:** Elements of a tenure-track faculty job offer and startup package that you can consider negotiating

	**Description**	**Considerations**
**APPOINTMENT**		
Contract or Service Period	Faculty appointments can have different terms. For example, they can be 9-, 10-, or 12-month appointments	These terms will affect how much base salary you receive The offer should specify how long is the initial appointment and when or how often it is renewed
Proportion of core responsibilities: research, teaching, and service	Most tenure-track faculty are expected to engage in three core types of activities– research, teaching, and service The amount of research, teaching, and service required and/or expected will vary depending on the institution	The offer should outline the general expectations for teaching and service (e.g., how many graduate and undergraduate classes per year, how much service, and which type). It's rare for service duties to be spelled out in detail in an offer, but general expectations for junior faculty are still useful to have in writing, especially because women and members of marginalized racial and ethnic groups often perform a disproportionate share of informal and formal service responsibilities. Some of this information will likely be found on the Faculty handbook for the institution or Departmental guidelines for faculty Depending on the institution, when the candidate transfers in funding and their associated indirect costs, they can often negotiate course releases or buy out teaching time to fulfill grant activities. For some grants to transfer, they must have a specific percentage of their time protected for research
Advancement Resources	Often, an institution provides new tenure-track hires with a faculty mentor or mentoring committee. These individuals can impact the new hire's success. Institutions may have additional advancement resources available	How are these faculty mentors or mentoring committee members chosen? What is their role in the career advancement of the new hire? What other faculty mentoring and advancement resources exist at the institution of interest?
**MONEY**		
Base Salary or Pay	This is the amount that an institution agrees to pay the faculty member per year. It should be listed in the offer letter	Will vary depending on whether a 9-, 10-, or 12-month faculty appointment For research-intensive institutions, an offer letter should stipulate what percentage of the faculty member's salary they are expected to recover from grants after the start-up period. It should also specify how summer salary will be covered
Professional Development (PD) Funds	Primarily Undergraduate Institutions can sometimes offer faculty funds each year for PD activities. Funds are institution-specific and can be in the range of $500-$2,500 At Research Intensive Institutions, professional development funds are not usually broken out separately from start-up money. In these cases, junior faculty can use their discretionary start-up money for a wide variety of professional development expenses	Allowed PD activities can vary but usually include society membership, scholarly travel, and technical courses
Research materials and equipment	Funds needed to get research projects started and off the ground	At research-intensive institutions, this money may be used to hire personnel such as staff (including overhead) or scholarly travel
**TIME**		
Time to spend start-up resources	Time given by the institution to use start-up resources	It can range from 1 year, at smaller institutions where start-up funds might be smaller, to 5 years. This time is negotiable The offer should specify whether unused start-up money is reclaimed or "taxed" by the school or retained entirely by the faculty member. Candidates can ask for the latter provision as part of the negotiations
Service release	The time during which the institution exempts faculty members from service responsibilities such as committee work and academic advising	New faculty are usually exempt from service duties during their first year at the institution
Teaching release	At institutions where there is a considerable teaching load, faculty can negotiate a course release or buy time out of teaching to have more time to give their research a strong start	If at a PUI, it might not be in your best interest to ask for a teaching release early on, given that the focus of the institution is teaching. Consider asking for a release if you are bringing grant money with you that requires a considerable amount of effort
Time to tenure review	Under certain circumstances, when a faculty member transfers from one institution to another without tenure, faculty members might be in the position to determine the timeline with which they come up for tenure	Knowledge of the institutional guidelines for tenure and promotion and a realistic assessment of where the candidate is with respect to meeting tenure and promotion requirements is needed to ensure success
**SPACE, EQUIPMENT AND SCIENTIFIC EXPERTISE**		
*Candidates should negotiate politely but firmly for the space they need BEFORE signing an offer. Their leverage evaporates as soon as the offer is signed, so no one should plan to adjudicate space concerns after accepting a position*		
Office space	The offer should include access to office space for the faculty member as well as their lab members. If at a PUI, office spaces for undergraduate student researchers are unlikely to be available or included. The office should come with office furniture and there might be funds for you to purchase office furniture needed. If funds are limited, institutions often have access to a warehouse where surplus furniture and resources might be stored. You can ask for access to this as needed. The surplus stored there might also include lab equipment	At this stage, it will help to have an idea of how big of a lab the candidate needs to have. The location of the office(s) with respect to the lab space(s) might be an aspect that is negotiable. Considering the campus location of potential collaborators or peers in the department might be advantageous to have offices neighboring them
Lab space	The offer should include access to a lab space for the faculty and their research team. The lab space might come with desks, so the lab members might not need a dedicated office space	Ensure the lab space is outfitted with the appropriate capacities, such as power and ventilation. It is also important that lab members have access to a place to eat meals and build community near the lab, especially if dedicated office spaces are not provided for them The offer should include square footage (and, preferably, specific room numbers) of the faculty member's lab space Ideally, the offer will explicitly state that the costs of any lab space renovations needed to accommodate the faculty member's research will be borne by the institution and will not come out of the faculty member's start-up funds. It is not uncommon for renovations to be necessary for scientific reasons (e.g., to accommodate a large instrument or to install vibration-dampening or light-blocking features). These expenses can be a major drain on the faculty member's start-up if they are forced to pay themselves
Space for your specialized equipment or research needs	A microscope room, cell culture room, or cold room. Negotiation for rooms to house specialized equipment might be less of an option at a PUI, where research space is more limited	The candidate should ensure that specialized needs for space to house equipment are taken into account. The space should be outfitted with the appropriate capacities, for example, power and ventilation
Access to shared equipment and/or core facilities	At research-intensive institutions, there will likely be a number of research core facilities that the faculty member and their lab members will have access to. Negotiation time is a great time to find out the limitations or constraints that come with the use of those resources and explore the possibility of trying to maximize them	Many times, at PUIs, most of the research-grade equipment is shared and available for students to use as they train as scientists. Institutions might post a list of equipment on their webpages or you can request this information to take into account as you plan and/or negotiate There might be surplus lab equipment that the institution has that is available to the new faculty member
Offering a specific resource	Resources such as funding, faculty expertise, facilities, and technology help universities reach their goals by enabling them to provide quality education, conduct impactful research, attract top students and faculty, foster a supportive learning environment, and ultimately achieve their mission of academic excellence and societal contribution	If a candidate has equipment, skills or expertise that can be viewed as a resource by their colleagues, the candidate should research the value or demand for that resource within the department. They should be able to communicate their understanding of its potential impact on the institution’s goals, and use that knowledge to negotiate salary and responsibilities

### If there are non-negotiables, respect them and use them as a way to better understand the institution

The individual negotiating on behalf of the institution might mention area(s) that are non-negotiable areas. In these cases, they will offer a rationale for these being non-negotiables. The candidate might have non-negotiables as well. If the institution discloses non-negotiables, this might be a good time for the candidate to disclose theirs. If the institution does not offer a rationale for the non-negotiables, it is acceptable to ask follow-up questions to reach a better understanding of what the institution’s limitations are. If you have non-negotiables, you might expect the institution representative to ask follow-up questions to understand your situation or needs better.

### Getting it in writing

When considering a job offer, verifying its legitimacy is a critical step that necessitates careful consideration. The only credible evidence of a job offer is an official, signed, written document that unambiguously defines the terms of the appointment, such as those summarized in Table 1. By taking these measures, an individual can ensure that they clearly understand the terms and conditions of their employment. Everything must be negotiated to the candidate's satisfaction and be included in the offer letter before signing. Once the offer letter has been signed, the candidate’s leverage evaporates.

### Body language and tone

While negotiation conversations can occur on the phone, it is becoming more common to have them through video conferencing. In these situations, experienced negotiators can interpret body language and utilize this as a source of information during negotiations. While body language can vary depending on an individual’s identity and culture, once you understand someone’s baseline behaviors, it is possible to gauge how your requests and ideas are being received [11]. If the conversation is over the phone, similar information may be obtained by paying close attention to someone’s tone–word choices, volume, and speed. The candidate should pay attention to the way the negotiation is handled by the institution and its representative. For example, was the process handled with respect and intentionality? This is information that can be used by the candidate to inform their decision of whether to accept the offer or not.

### Making a decision

#### Accepting an offer

Job offers from academic institutions are increasingly being presented as legally binding contracts. These contracts contain detailed information about the job, including language related to promotions, background checks, appointments, and employment eligibility. There are many examples available online. If an individual accepts such an offer, they must consider the reasons behind the decision, weigh the options carefully, and ensure that the decision aligns with their career goals. Before signing and submitting the document to the employing institution, the candidate needs to review and confirm all the terms and conditions offered in the contract. If there are details from the negotiations that were overlooked, the candidate can ask to continue the conversation and ask for changes or revisions to the letter. If the applicant would like to accept an offer, it is recommended that this is done by phone and then followed up with the signed contract to confirm their decision. It is vital to note that once the contract is signed, the candidate is ethically obligated to fulfill their commitment under the agreed-upon terms. Additional resources can be found in books that focus on academic job search [12] and those that discuss specific considerations for scientists [13].

#### Declining an offer

Declining a job offer is a task that requires a level of finesse and is an essential skill to learn. It is critical to maintain good relationships and avoid burning bridges. Various reasons may warrant the rejection of an offer. These include the position being unsuitable or not a good fit, an inadequate compensation package, life changes, a red flag being raised during the negotiation, or the reception of a more desirable offer. Regardless, the employment opportunity must be declined gracefully to preserve one's reputation. Therefore, it is crucial to approach the task of declining a job offer with a measure of professionalism, tact, and diplomacy. Before declining an offer, it is essential to consider the reasons behind the decision, weigh the options carefully, and ensure that the decision aligns with one's career goals. A well-crafted email expressing gratitude for the consideration and interest in the position should be written. The email should also convey a polite and respectful tone while stating the reasons for declining the offer. Candidates do not have to provide a detailed explanation of why they are declining an offer if they prefer not to do so. It is sufficient to express gratitude for the offer and simply state that another offer or opportunity proved to be a better fit. Ultimately, the process should preserve the relationship between the parties involved and demonstrate a commitment to ethical and professional conduct. Additional resources can be found in books that focus on faculty job searches for scientists [14].

## Conclusions and final remarks

As candidates get ready to negotiate the terms of their faculty position, it is worth remembering that this is also the perfect opportunity to get to know their potential future employer. The consideration, intentionality, and interest they display in setting the candidate up for success is likely a strong indicator of how they will be treated once they are a faculty member there. If they are unable to secure a start-up package that meets their basic needs and expectations, being prepared to walk away will ultimately open the door to finding an institution that is a better match. In Table 2, we include a list of *Frequently asked questions on startup package negotiation.* Additional resources that will likely be useful when negotiating a job offer include articles written by other scientists [15–27]. There are other resources that highlight the institutional perspective of the job search, including the negotiation process [28]. When it comes to negotiating the terms of a faculty position, there are various effective strategies that can lead to a positive outcome. Negotiations can help set up the new faculty member for success, including the attainment of tenure and promotion. However, it is important to note that the candidate themselves are ultimately responsible for selecting the details of the negotiation process that are best suited to their needs. The skills developed during the negotiation process can be useful throughout a faculty member's career progression [29].

**Table 2:** Frequently asked questions on startup package negotiation. For many of these questions, the right answer is specific to the individual’s circumstances. Here, we provide considerations for candidates to inform their decision-making

	**Question**	**Considerations**
**PREPARATION**		
Defining needs	When should the candidate start thinking about what they need to include in their start-up package?	The earlier the candidate starts thinking about their needs, the more time they will have to refine and perfect their list
Defining job search preferences	Should I keep my job search process secret?	If the candidate *needs* to keep their job search discreet, they may prefer to conduct it confidentially and seek feedback and reference letters from sources other than their direct supervisor The candidate should consider the potential consequences if their current supervisor were to find out about their job search
Defining negotiables	Is it a deal breaker if there is a lack of 'good fit' with the department?	Having a supportive environment is crucial for career progression as a faculty member. Scientific fit can contribute to creating such an environment
**MONEY & GRANTS**		
Base Salary or Pay	If you work for a public (vs. private) university, how much can you usually negotiate a higher salary at hiring?	Faculty appointments can have different terms, such as 9, 10, or 12 months, which will affect monthly salary The answer to this question is discipline- and institution-specific. The candidate must approximate the average salary using resources like knowledgeable colleagues or publicly available salary data online
	Different departments within the same university offer different salaries for the same position. Could this mean there's room for negotiation to increase the initial salary in a specific department?	
	If the initial salary wasn't negotiated well, what are the options for getting a salary increase? Is a promotion the only way, especially since promotions might only happen every five years?	It is common for institutions to audit faculty salaries every few years to ensure equity. Faculty found to be below the salary benchmark for their position are raised to an equitable range During the negotiation process, the candidate can ask about the institution’s faculty compensation audits
Grants	If I bring a grant(s) with me to my new position, how should I best leverage those resources during the negotiation process?	Depending on the institution, when the candidate transfers in funding and their associated indirect costs, they can often negotiate course releases or buy out teaching time to fulfill grant activities Some fellowships have requirements that should be addressed in an offer letter. For example, the NIH K99/R00 fellowship requires a statement in the offer letter that 75% of the faculty member's time will be protected for research. This is a precondition for activating the R00 phase of the award
Start-up amount	What's the average amount for start-up packages that one should negotiate for?	The average amount will vary per institution. In order to prepare for the negotiation process, the candidate will have to investigate to determine the approximate value of start-ups at the institution of interest The amount negotiated should be driven by scientific need. A synthetic organic chemist likely needs less start-up money than a geneticist with a large mouse colony, so differences in start-up amounts (even within a single department) are not necessarily inequitable
**NEGOTIATION DYNAMICS**		
Who should be part of the process	Who should be present during negotiation meetings?	At the very least, the candidate and the individual that the institution has charged with negotiating should be present
Developing confidence	How do you generate the confidence to negotiate without feeling like an imposter?	Preparation is needed to enter the negotiation phase with confidence It is key to remember that the candidate would not be this far in the application process if they were not what the institution is looking for in a faculty member. The candidate must trust their abilities! If an offer has been made, the department really wants to hire the candidate. Candidates should negotiate vigorously and confidently, knowing that the institution wants to recruit them and set them up for success
**SPACE**		
	If the initial space allocation wasn't negotiated well or turned out to not be enough, what are the options for getting more space?	It is usually advisable to follow the chain of command and ask the chair of the department for assistance in obtaining additional space. The faculty member should be prepared to justify the need for such space. If the faculty member has joined a supportive environment that is invested in their success, they will find a way to assist and alleviate the needs of the faculty member

## Data Availability

Not applicable.

## Abbreviations

ACT: Accomplishing Career Transitions
ASCB: American Society for Cell Biology
AAUP: American Association of University Professors
BMC: BioMed Central
HUE: Historically underserved and excluded groups
NIH/NIGMS: National Institutes of Health/National Institute of General Medical Sciences
PI: Principal Investigator
PUIs: Primarily undergraduate institutions
PD: Professional Development
STEM: Science, Technology, Engineering, and Math
